# Medical Cannabis for Intractable Epilepsy in Childhood: A Review

**DOI:** 10.5041/RMMJ.10387

**Published:** 2020-01-30

**Authors:** Bruria Ben-Zeev

**Affiliations:** 1 Pediatric Neurology Department, The Edmond and Lilly Safra Pediatric Hospital, Sheba Medical Center, Tel Hashomer, Israel; 2 Sackler School of Medicine, Tel Aviv University, Tel Aviv, Israel

**Keywords:** Artisanal cannabis, CBD, intractable epilepsy, Dravet syndrome, Lennox–Gastaut syndrome

## Abstract

In recent years, cannabis has been gaining increasing interest in both the medical research and clinical fields, with regard to its therapeutic effects in various disorders. One of the major fields of interest is its role as an anticonvulsant for refractory epilepsy, especially in the pediatric population. This paper presents and discusses the current accumulated knowledge regarding artisanal cannabis and Epidiolex®, a United States Food and Drug Administration (FDA)-approved pure cannabidiol (CBD), in epilepsy management in pediatrics, by reviewing the literature and raising debate regarding further research directions.

## INTRODUCTION

Childhood epilepsy may be coarsely divided into (1) a large group (70%) of benign epileptic syndromes and easily controlled symptomatic epilepsies, and (2) a smaller but significant group of drug-resistant epilepsies which includes idiopathic and genetic epileptic encephalopathies and various symptomatic acquired epilepsies. The burden of intractable epilepsy on infants and children and their families is enormous, and in addition to the risks carried by the actual seizures it significantly affects the children’s development and quality of life. Most of these children are on polytherapy, which has its own consequences. This devastating situation has led to a quest for additional solutions. This quest, with regard to the use of cannabis, has been led by parents and caretakers in parallel to the medical authorities.

Various ancient cultures have mentioned cannabis as a useful tool to treat epileptic convulsions. There are historical records from ancient China dating back to 2700 BC^1^ and tablets written by the Sumerian and Akkadian peoples in 1800 BC,^2^ as well as other ancient historical records. In the nineteenth century several leading physicians published papers on its use as an anticonvulsant, presenting both case reports^3^ and their general impression on its effectiveness when added to bromides.^4^

Despite the Marijuana Tax Act of 1937, which led to the removal of cannabis from the US pharmacopeia in 1941, and its classification as a schedule 1 substance, several researchers and physicians renewed investigation on the biological effects and medicinal use of its various components in the 1970s. Several animal studies and small-scale clinical trials examined its use. Studies focusing mainly on purified cannabidiol (CBD) in epilepsy management in drug-resistant patients were published.^5^–^10^ The above clinical studies were assessed in a 2012 Cochrane review stating that the trials were based on small samples with inconsistent products, dosages, dose frequencies, and treatment durations. These deficiencies led the Cochrane reviewers to conclude that CBD efficacy in the treatment of epilepsy could not be confirmed, but that a dosage within the range 200–300 mg daily was safe enough to be given over a short time period.^11^

In the last decade social media, patient and family advocacy groups, and Internet activity have led to significant public interest in cannabis as an alternative treatment for those living with epilepsy. This public demand revived both basic and clinical research into cannabis and CBD use for epilepsy treatment particularly in the pediatric population. This review provides an overview and discussion of the current data relating to artisanal cannabis and pure CBD use in epilepsy.

## CANNABIS AND CANNABIDIOL ANTIEPILEPTIC MECHANISM OF ACTION AND PHARMACOKINETICS

The marijuana plant, *Cannabis sativa*, and *Cannabis indica* contain up to 500 chemical species, with more than 100 different phytocannabinoid compounds.^12^ The two main components of cannabis—Δ-9-tetrahydrocannabiniol (THC) and CBD—have generated the most interest in terms of their putative effectiveness as anti-seizure agents: THC is a psychoactive agent, with equivocal value for seizure control and a potential to trigger seizure activity; CBD is a non-psychoactive agent with both anecdotal and scientific evidence suggesting its usefulness as an antiepileptic medication.^13^,^14^

Biologically, THC’s mechanism of action is primarily related to its effect on endogenous cannabinoid receptors in the brain, mainly cannabinoid receptor type 1 (CB1), and to the extra central nervous system (CNS) receptor, CB2.^15^ Cannabidiol, on the other hand, has a relatively small direct affinity to the CB1 and CB2 receptors but has an inhibitory effect on THC binding to the CB1 receptors.^16^ This inhibitory effect may lead to positive modulation (“fine tuning”) of CB1 activation by THC which may reduce anxiety and paranoia caused by its non-modulated activation.^17^

The CBD anticonvulsant activity is most probably multifactorial and relates to: gamma-aminobutyric acid (GABA)-mediated inhibition of glutaminergic forebrain neurons^18^; intracellular calcium current modulation through an effect on several transient receptor potential channels of the vanilloid subtype; its direct effect on the G-protein-coupled receptor GPR55; and the inhibition of adenosine reuptake and modulation of tumor necrosis factor (TNF)-alpha release, affecting the inflammatory related components of epileptiform activity.^19^ The affinity of CBD for the 5-HT1A and 5-HT2A receptors is also considered a novel target for refractory epilepsy treatment.^20^ In addition to its indirect antagonism on CB1, CBD may affect the seizure threshold as shown in several animal studies.^21^

## CANNABIDIOL ANTICONVULSANT EFFECTS IN ANIMAL MODELS

Cannabidiol has been tested in several animal epileptic models, including maximal electroshock, pentylenetetrazol, pilocarpine, penicillin, audiogenic seizures, 6-Hz, subcutaneous metrazol threshold test, and cobalt implantation,^22^–^26^ and was found to have an anticonvulsant effect in all models. Its anticonvulsant profile was re-evaluated using the focused screening protocol developed by the National Institute of Neurological Disorders and Stroke (NINDS)-funded Epilepsy Therapy Screening Program. Intraperitoneal introduction of CBD produced a dose-dependent protection against maximal electroshock-induced seizures in mice and rats and was found to be effective in the 6 Hz, 44 mA seizure model and the corneal kindling model in mice.^27^

Because of the specific interest given to the positive effect of CBD in Dravet syndrome patients, this compound was studied in an SCN1A knockout mouse model showing decreased spontaneous seizure frequency and duration, as well as decreased severity of heat-induced seizures. Autistic-like social interaction deficits improved with low-dose CBD but failed to improve with the higher dosages required for seizure control.^28^

Studying the effect of THC on seizures in various animal models showed conflicting results—including anticonvulsant, no effect, and proconvulsant responses—making it less attractive for clinical epilepsy treatment.^29^

## PHARMACOKINETICS

Cannabidiol has poor oral bioavailability of around 6%, which is related to its lipophilic structure, variable absorption rate, and extensive hepatic first-pass metabolism by isozymes CYP2C19 and CYP3A4. Its bioavailability can be increased or decreased by exposure to a strong enzyme inhibitor or inducer, respectively.^30^ It is highly protein-bound and because of its lipophilic structure may accumulate in adipose tissues. The CBD peak plasma concentrations after oral administration in oily formula is about 2.5 hours,^31^ with its biphasic elimination (initial half-life of 6 hours and terminal half-life of 18–32 hours) reflecting distributive processes into tissues.^32^

## CURRENT CLINICAL EXPERIENCE

Over the last six years, medical publications regarding epilepsy treatment with cannabis oil extracts and pure CBD can be divided into several groups: retrospective surveys and chart reviews of patients independently treated with artisanal cannabis by their caretakers which was reported to physicians; retrospective chart reviews of CBD-enriched cannabis oil use, as directed by physicians; and open-label followed by placebo-controlled prospective studies of US Food and Drug Administration (FDA)-approved pure CBD oil (Epidiolex®); in addition to anecdotal reports of other “pure CBD” compounds used.^33^

Porter et al. surveyed parents who belong to a Facebook group that used CBD extracts to treat their children’s seizures. Nineteen out of 150 participants in the group responded: 84% reported a reduction in seizure frequency; of these, 11% (2/16) experienced a positive effect and became seizure-free.^34^ Another online survey on CBD extract effect published by Hussain et al. included responses from 117 parents of children with epilepsy (more than 40% with intractable epileptic encephalopathies); the parents reported 85% responders, with 14% of the children achieving seizure freedom.^35^ A third internet survey was reported from Mexico,^36^ describing 53 patients, age range 9 months–18 years, using various cannabis extract compounds, mostly of unknown composition: 83% of patients experienced improved seizure control, and 16% became seizure-free, with only a 5% rate of seizure aggravation. However, for all these publications there is selection bias of the reports, and additionally the extract used, reported dosages, and lengths of treatment were inconsistent.

A retrospective medical chart review was performed on 75 children and adolescents who received various types and dosages of oral cannabis extracts to treat their epilepsy. Based on parental reports, within the subgroup of responders (33%), more than a 50% reduction of seizures was noted in children with Lennox–Gastaut syndrome (LGS) and Dravet syndrome. Interestingly there was a significantly higher response rate from families who had moved to Colorado in order access artisanal cannabis treatment for their child as compared to the families who were already Colorado residents (47% versus 22%), suggesting a higher placebo effect in those with higher treatment expectations.^37^

A retrospective multicenter study was performed with data from three epilepsy clinics in Israel treating 74 children for intractable epilepsy with one of two well-controlled cannabis oil extracts (CBD:THC ratio, 20:1; dosage range 1–20 mg/kg/d for 3–12 months). The authors found that 52% of patients experienced more than 50% reduction in seizure frequency; only 7% of patients experienced seizure aggravation.^38^ This study differs from the previous ones in that the cannabis oil treatment was directed by the pediatric neurologists responsible for all patient treatment decisions during the follow-up period. The largest chart review published so far on the effect of artisanal cannabis in pediatric epilepsy is by Sulak et al., reviewing information on 272 pediatric patients from Washington and California states who were followed for 3–30 months. They noted a more than 50% reduction in seizure frequency in 45% of patients, with 10% becoming seizure-free.^39^

While the use of artisanal cannabis preparations can be criticized as being inaccurate and not as precise as expected for other drug treatments in the medical community, a more well-controlled process leading to FDA and European Medicines Agency (EMA) approval was performed for a pure CBD extract produced by GW Pharma called “Epidiolex.”

The first published study using Epidiolex as an add-on treatment for children with intractable epilepsy was by Devinsky et al.^40^ This open-label multicenter efficacy and safety study included 214 patients (aged 1–30 years) with severe childhood-onset, drug-resistant epilepsy (33 patients had Dravet syndrome; 31 patients had LGS). A 36.5% median reduction in monthly motor seizures was found in the 137 patients eligible for analysis.

The following study^32^ was already a double-blind placebo-controlled trial looking at the effectiveness of CBD oil (Epidiolex) as an add-on agent compared with placebo in 120 children and adolescents with treatment-resistant seizures related to Dravet syndrome (CBD dosage 20 mg/kg/d). After a four-week titration period, patients were followed for an additional 12 weeks. The main significant effect noted was the rate of reduction in convulsive seizures between CBD-treated patients (43% response rate) and the placebo group (27%), with 5% becoming seizure-free in the treatment versus placebo groups. The difference between groups for non-convulsive seizures was not significant. There was a larger drop-off (15%) in the treatment arm, compared to placebo (5%), as well as a higher rate of side effects (93% versus 85%). The response rate was higher in patients adding CBD to clobazam.^32^,^41^

Two multicenter double-blind placebo-controlled trials investigated the short-term effect of CBD in LGS patients: the first one looked at the efficacy of CBD (20 mg/kg/day) as an add-on therapy for drop seizures in 171 patients (2–55 years) with treatment-resistant LGS and found a statistically significant reduction in drop seizure number in the CBD compared to the placebo group.^42^ The second study compared two doses (20 mg/kg/day and 10 mg/kg/day) of purified CBD to placebo and demonstrated a significant reduction in drop seizures versus placebo with both dosages.^43^

Equally important to double-blind placebo-controlled studies, which are usually short term, is investigation into a drug’s long-term effect on epilepsy. This collected data was lately reported by Szaflarski et al.^44^ who reviewed the safety data of 607 patients, and the efficacy data of 580 patients, participating in the extended protocol Epidiolex studies. They found that the improvements experienced during the first 12 weeks of the study were sustained over the 96 weeks of the study, in most of the patients available for this analysis, with no aggravated side effects. The 76% study retention rate compares favorably to other antiepileptic drug trials.

Based on these positive trials the FDA approved pharmaceutical-grade CBD Epidiolex as an oral solution (100 mg CBD/mL) for treating seizures in LGS and Dravet syndrome patients over the age of two years. However, other CBD products remain as schedule I substances under the Controlled Substances Act. The above-mentioned approval was followed by EMA approval of Epidiolex for the same indications and age group, but only when added to clobazam, based on the assumption that because CBD increases blood levels of clobazam active metabolite, its antiepileptic effect in many patients in the randomized studies that led to the FDA approval could be attributed to this effect only.

Smaller-scale studies looked at the effect of Epidiolex in other rare epileptic syndromes and disorders with severe epilepsy, including its positive effect on seizures in tuberous sclerosis patients^45^; a higher response rate was noted when CBD (Epidiolex) was added to clobazam. A promising effect was also reported in an open-label study in 6/7 patients with febrile infection-related epilepsy syndrome,^46^ as well as in Sturge–Weber syndrome.^47^

## CANNABIDIOL INTERACTIONS

Cannabidiol is a potent inhibitor of CYP2C19, CYP2D6, and CYP2C9, which leads to an increase in the level of several antiepileptic drugs, with the most significant effect being on clobazam and its metabolite N-desmethylclobazam, and a less prominent effect on topiramate, eslicarbazepine, zonisamide, rufinamide, and brivaracetam.^48^–^50^

Abnormal elevation in liver enzymes (transaminases) can occur with concomitant use of valproate and CBD without significant changes in the valproate levels, suggesting a pharmacodynamic rather than a pharmacokinetic interaction.^32^

## CANNABIDIOL AND CANNABIDIOL-ENRICHED CANNABIS ADVERSE EVENTS AND POSITIVE NON-EPILEPSY-RELATED EFFECTS

The adverse events reported in the well-controlled trials of both artisanal cannabis use and Epidiolex were qualitatively similar and included fatigue, decreased appetite, somnolence, vomiting, diarrhea, and seizures. Somnolence was more frequently reported in patients treated with Epidiolex in addition to clobazam.^47^,^48^ In the extensive chart review by Sulak et al., related to artisanal cannabis use, the main side effects were somnolence and fatigue in up to 20% of patients.^39^ The same side effects with similar or slightly higher rates were reported in the other artisanal cannabis studies,^34^,^38^ while various Epidiolex studies showed a significantly higher side effects rate. In the open-label study by Devinski et al.,^40^ a 79% adverse event rate was reported, with 25% somnolence, 19% decreased appetite, 19% diarrhea, 13% fatigue, and 11% convulsions; 3% of patients discontinued treatment because of an adverse event. Serious adverse events as defined by the research protocol were reported in 30% patients; in 12% these effects were possibly related to cannabidiol use, the most common of which was status epilepticus (6%). For LGS treatment, a comparison study of two CBD dosage groups, 10 mg and 20 mg, reported adverse events in 84% and 94%, respectively, but a significant adverse event rate of 72% was also reported in the placebo arm. The investigators judged 89% of the events to be of mild or moderate severity. The most common adverse events were, again, somnolence, decreased appetite, diarrhea, upper respiratory tract infection, pyrexia, and vomiting.^43^

Elevations of liver transaminases were reported only in the Epidiolex studies, mainly during the first two months after treatment initiation, and were primarily dose-related; delayed transaminase elevations have also been noted, particularly with concomitant valproate use, and less frequently with concomitant clobazam use. The transaminase elevation was reversible with discontinuation or reduction of CBD oil and/or concomitant valproate.^32^

The significantly higher rate of adverse events reported in the Epidiolex well-controlled trials compared to the open-label artisanal cannabis reports may be partially related to bias effect of the artisanal retrospective reports; the high rate of adverse events reported in the placebo group of the Epidiolex studies supports this assumption. But it could also be related to the much higher dose and rapid titration rate of CBD used in the Epidiolex studies as compared to artisanal cannabis use. The possibility that differing amounts of other constituents in artisanal cannabis oil, such as THC, other cannabinoids, and terpenes, have a protective effect merits further investigation.

It is interesting to note that the artisanal cannabis reports and the short- and long-term Epidiolex studies reported other positive side effects not directly related to seizure control. The beneficial effects of CBD-enriched cannabis, other than reduced seizures, were reported in all studies related to artisanal cannabis use and included: increased alertness (>50%); improved sleep (25%–68%), behavior (33%), language (10%), and motor skills (10%–20%); and decreased self-stimulation (32%).^34^,^35^,^37^–^39^ Although there is no specific reference to these effects in the Epidiolex studies, a recent report on quality of life (QoL) in pediatric patients enrolled in a CBD (via Epidiolex) study has shown significant improvement in caregiver-reported QoL in multiple domains, as well as in general. This may be related to both better seizure control as well as additional positive changes noted in the patients’ conditions.^51^

## THOUGHTS, PRECAUTIONS, AND CONCLUSIONS

Pure CBD (i.e. Epidiolex) and CBD-enriched cannabis oil extracts were found to be effective for epileptic seizure control in pediatric patients and young adults, particularly in the specific epileptic syndromes, Dravet syndrome and LGS, as was the case in larger more varied groups of patients with intractable epilepsy. Based on the current data, it is essential that drug formulations contain as low a THC content as possible, since the anti-seizure activity of THC is equivocal and it can potentially aggravate seizures; moreover, it can be associated with additional short- and long-term side effects, especially related to memory as well as other aspects of cognition and behavior.^52^

We should be aware that artisanal and other commercially available products are currently not well controlled. This has been proven through in-depth chemical profiling of cannabinoids, terpenes, and oxidation products of commercially available CBD oils used for treating epilepsy in the United States, which found that 9/14 of the samples studied (64%) had concentrations that differed from the declared amount, with only five maintaining optimal concentrations.^53^ Strict regulations for manufacturing, packaging, and labeling are warranted to ensure safe administration and efficient use of cannabis extracts, and would enable wider use for treatment of intractable epilepsy in the pediatric and possibly the adult population.

On the other hand, future research on the role of cannabis in epilepsy should keep in mind that the controlled, randomized trials have revealed that the actual reduction in seizure frequency in response to CBD is comparable to that achieved in response to other antiepileptic drugs, and have failed to meet the 60%–85% responder rates in unblended web-based surveys and chart reviews based on parental reports. In addition the rate of side effects was higher in the well-controlled Epidiolex studies compared to artisanal cannabis use. Although these differences may primarily be related to the positive bias of the open-label retrospective studies and higher CBD dosages used in the controlled pure CBD trials, there is still room for debate regarding the role of other phytocannabinoids present in artisanal cannabis extracts, which may have an “entourage effect” on both the anticonvulsant potency and their protective role, which points to the need for further research in this direction.^54^

## Abbreviations

CB1: cannabinoid receptor type 1
CB2: cannabinoid receptor type 2
CBD: cannabidiol
CNS: central nervous system
EMA: European Medicines Agency
FDA: United States Food and Drug Administration
LGS: Lennox–Gastaut syndrome
NINDS: National Institute of Neurological Disorders and Stroke
QoL: quality of life
THC: Δ-9-tetrahydrocannabiniol.
